# Alert to Hurt: Threat Appraisal Mediates the Association Between Attachment Anxiety and Perceptions of Pain Burden Among Healthy Young Adults

**DOI:** 10.3390/bs16091579

**Published:** 2026-09-07

**Authors:** Trevor C. J. Shannon, Lauren E. Cravens, Richard S. Pond

**Affiliations:** Department of Psychology, University of North Carolina Wilmington, Wilmington, NC 28403, USA; trevorshannon@yahoo.ca (T.C.J.S.); lec1781@uncw.edu (L.E.C.)

**Keywords:** attachment anxiety, threat appraisal, alexithymia, pain

## Abstract

The experience of pain can vary considerably from one person to the next. Consequently, research has begun to explore the factors that may predict this inter-individual variability, including individual differences in attachment anxiety. Attachment anxiety has been linked convincingly to sensitivity to pain and susceptibility to chronic pain disorders. Still, the leading model relevant to the relationship between attachment anxiety and pain is highly complex and may not generalize to populations that do not, specifically, suffer from chronic pain. In the current study, we build off this past literature and theorized that threat appraisal and alexithymia, which are each closely related to attachment anxiety, will explain the relationship between attachment anxiety and perceived pain burden among healthy young adults. Results partially supported our primary mediation hypotheses, such that both threat appraisal and alexithymia each exhibited a significant indirect effect underlying the association between attachment anxiety and perceived pain burden. However, only threat appraisal yielded a significant indirect effect in a parallel mediation model. Our mediation findings provide encouraging insights into the relationship between attachment anxiety and the pain experience.

## 1. Introduction

Over the past three decades, accumulating evidence has highlighted the role of both biological and psychosocial factors in shaping how individuals experience, cope with, and accept pain (e.g., Carneiro et al., 2025; for a review, see Fillingim, 2017). One psychosocial factor that has garnered increasing attention is attachment anxiety–an interpersonal orientation characterized by heightened concerns about rejection and abandonment by close others (Bretherton, 1992). Attachment anxiety, in turn, has been consistently associated with the experience of pain (e.g., P. Meredith et al., 2008; McWilliams & Bailey, 2010; for reviews, see Failo, 2022, and Stamp et al., 2024).

Despite mounting research on the variables that may mediate the relationship between attachment anxiety and pain (Laird et al., 2015; P. Meredith et al., 2008; Peñacoba et al., 2018), it remains unclear which is most essential. Furthermore, most of the literature concerning the association between attachment anxiety and pain was conducted within the context of those who suffer from chronic pain conditions (Failo, 2022; Stamp et al., 2024). The aim of the current study was to isolate the variables driving the perceived burden of pain among young adults with high levels of attachment anxiety, outside of the context of a chronic pain diagnosis. Specifically, we proposed that threat appraisal and alexithymia would serve as mediating mechanisms linking attachment anxiety to perceived pain burden. Threat appraisal reflects the belief that one is unable to meet the demands posed by one’s pain (Lazarus & Folkman, 1984), whereas alexithymia refers to an externally-oriented cognitive style involving difficulties in identifying and communicating feelings and distinguishing those feelings from somatic sensations (Taylor et al., 1991).

### 1.1. The Experience of Pain

Pain represents one of the most common reasons individuals seek medical care, accounting for as many as 80% of physician visits (Voscopoulos & Lema, 2010). Yet, the experience of pain is far from uniform, with considerable variability observed across individuals exposed to similar painful stimuli (Coghill et al., 2003). Although the processes responsible for such variability are not fully understood, pain perception is believed to emerge, in part, from the integration of afferent sensory input with expectations regarding the painful stimulus (Koyama et al., 2005; Posner et al., 1980). Importantly, expectations and incoming sensory information do not always correspond, and discrepancies between the two may contribute to altered pain experiences and, potentially, the development or persistence of conditions such as chronic pain (Koyama et al., 2005).

The consequences associated with persistent pain are substantial. Estimates suggest that pain conditions affect more than 100 million adults in the United States and place a considerable burden on the healthcare system (Gaskin & Richard, 2012; Institute of Medicine, 2011). Their broader societal costs are similarly significant, encompassing losses in workplace productivity and tax revenue, as well as expenditures related to disability compensation and legal services (Dansie & Turk, 2013). Compounding these costs, many individuals experiencing significant pain fail to obtain adequate relief with available treatments (Dansie & Turk, 2013). Together, the high prevalence of pain, its considerable individual and societal consequences, and the limitations of existing approaches to pain management highlight the importance of identifying factors that increase vulnerability to burdensome pain experiences. Such knowledge may ultimately inform the development of preventative interventions aimed at individuals who are particularly susceptible to experiencing adverse pain outcomes.

### 1.2. Attachment Anxiety and Pain

Attachment anxiety has been consistently tied to susceptibility to pain, including an increased sensitivity to pain (Shannon & Pond, 2022), increased pain intensity and disability (Andersen et al., 2026; Borthwick et al., 2024) and specific factors that may predispose individuals to developing chronic pain (McWilliams & Bailey, 2010). Attachment anxiety may heighten vulnerability to chronic pain through characteristic patterns of responding to painful experiences. Specifically, individuals high in attachment anxiety tend to engage in greater pain catastrophizing, report lower pain self-efficacy, perceive their pain as more severe, and experience greater pain-related psychological distress—responses that are consistent with the heightened activation of the attachment system and efforts to obtain reassurance and support from close others (P. Meredith et al., 2008; Porter et al., 2007). Thus, attachment anxiety may represent more than a correlate of heightened pain vulnerability; it may also contribute to patterns of appraisal and responding that amplify acute pain experiences and increase the likelihood that pain persists over time (McWilliams & Bailey, 2010; P. Meredith et al., 2008; Porter et al., 2007; Stamp et al., 2024).

### 1.3. Possible Mediators of the Attachment Anxiety and Pain Relationship

Growing attention has been devoted to identifying the psychological processes that may account for associations between attachment orientation and pain experiences (Romeo et al., 2017). Central to this work is the Attachment-Diathesis Model of Chronic Pain (ADMCP; P. Meredith et al., 2008), which provides a theoretical framework for understanding how attachment orientations may shape individuals’ experiences of and adjustment to pain. According to the ADMCP, attachment orientations influence how individuals appraise pain, evaluate their own ability to cope with pain and worthiness of receiving support, and perceive others as available and willing sources of support. These appraisals, in turn, shape coping and support-seeking behaviors, emotional responses, and ultimately adjustment to pain. From this perspective, psychological processes involving pain appraisal, self-efficacy, perceived social support, coping, support-seeking, and emotional responding may help explain why attachment anxiety is associated with poorer pain outcomes.

A growing, albeit still relatively limited, body of empirical research provides support for these proposed pathways. Among individuals higher in attachment anxiety, pain outcomes have been linked to threat appraisal (P. J. Meredith et al., 2005), anxiety (Peñacoba et al., 2018; Tremblay & Sullivan, 2010), anger (Liu et al., 2011), passive coping (i.e., coping characterized by inaction, maladaptive cognitions, and limited active problem-solving; Laird et al., 2015), pain beliefs involving lower pain self-efficacy and greater perceptions of pain threat (Laird et al., 2015), pain catastrophizing (Andrews et al., 2014; Kratz et al., 2012), pain-related fear (Martínez et al., 2012), self-perceived burden (Kowal et al., 2012), negative spousal responses to pain behavior (Forsythe et al., 2012), and activity avoidance (Andrews et al., 2014). Collectively, these findings are consistent with the ADMCP’s proposition that attachment anxiety may confer vulnerability to adverse pain experiences, at least in part, by shaping how individuals cognitively, emotionally, and behaviorally respond to pain.

Attachment anxiety has also been connected to pain through alexithymia (Aaron et al., 2019; Habibi Asgarabad et al., 2023; Peñacoba et al., 2018; Zdankiewicz-Ścigała & Ścigała, 2018), a factor unaccounted for by the ADMCP. This relationship is of note given the presumed etiology of alexithymia, which may parallel that of anxious attachment (i.e., influenced by disturbances in the caregiver-infant relationship, growing up in emotionally volatile environments; Berenbaum & James, 1994; Montebarocci et al., 2004). That is, alexithymia and attachment anxiety may share some developmental antecedents (see Taylor et al., 2024 for a review); however, it is important to consider that much of this research relies on retrospective reports from adult respondents.

Alexithymia reflects difficulties identifying, understanding, and communicating emotional experiences and interpreting the somatic states associated with emotional arousal (Bucci, 1997). Because these difficulties may interfere with the effective processing and regulation of physiological activation, individuals higher in alexithymia may experience more pronounced or persistent physiological responses to distress (Bucci, 1997; Taylor, 2000). Attachment anxiety may increase vulnerability to these alexithymic tendencies, thereby providing one potential pathway through which attachment-related difficulties contribute to somatic problems, including the experience of pain (Bucci, 1997; Habibi Asgarabad et al., 2023; Taylor, 2000).

Although numerous psychological processes have been implicated in the relationship between attachment anxiety and pain, considerably less is known about which processes may be most fundamental to this association. Clarifying these relationships is particularly important given the ADMCP’s proposition that many of the psychological factors involved in pain adjustment reciprocally influence one another (P. Meredith et al., 2008). Consequently, identifying more precise pathways through which attachment anxiety contributes to pain burden may help distinguish processes that play a more foundational role from those that emerge further downstream. Such distinctions could, in turn, inform more targeted interventions by identifying psychological processes that contribute to subsequent maladaptive pain-related cognitions and behaviors.

### 1.4. The Current Study

Though previous research has advanced our understanding of the experience of pain among individuals with high levels of attachment anxiety, it offers little insight into the principal factors driving connections between attachment anxiety and pain. Furthermore, the vast majority of studies within this literature concern sufferers of chronic pain (see Failo, 2022 and Stamp et al., 2024). Therefore, it is important to examine how well patterns in this literature generalize to populations without a chronic pain diagnosis. The current study’s aim was to address these issues by proposing a model for the relationship between attachment anxiety and the experience of pain among healthy young adults. As the ADMCP (P. Meredith et al., 2008) is the foundational model within this literature, we based our predictions upon the ADMCP. Specifically, we propose that only two mediators are needed to explain the various psychological factors proposed by the ADMCP.

Of the psychological processes proposed by the ADMCP, threat appraisal may be particularly fundamental to understanding the relationship between attachment anxiety and pain (P. Meredith et al., 2008). The model suggests that appraising pain as threatening can shape many of the subsequent cognitive, emotional, and behavioral responses associated with adjustment to pain. Moreover, heightened vigilance to threat is itself a central characteristic of anxious attachment (Bartholomew et al., 1997), providing a theoretical basis for expecting people high in attachment anxiety to interpret painful experiences as especially threatening. Consistent with this reasoning, accumulating evidence identifies threat appraisal as an important mechanism through which attachment anxiety may be associated with adverse pain experiences (Andrews et al., 2014; Chester et al., 2012; Martínez et al., 2012; P. J. Meredith et al., 2005; Stamp et al., 2024). We, therefore, predicted that greater attachment anxiety would be associated with greater perceived pain burden, in part through heightened threat appraisal.

Alexithymia may represent a second fundamental process underlying this association. This possibility is supported, first, by evidence suggesting that alexithymia and anxious attachment may share developmental antecedents (Berenbaum & James, 1994; Montebarocci et al., 2004; Taylor et al., 2024). Rather than representing only a consequence of maladaptive adjustment to pain, alexithymia may constitute a broader vulnerability that shapes how individuals subsequently interpret, regulate, and communicate painful experiences. Second, alexithymia is associated with a striking range of psychological and interpersonal processes that have themselves been implicated in attachment-related pain vulnerability, including substance-related coping (Hamidi et al., 2010), negative affectivity (Lumley et al., 2002; Makino et al., 2013), interpersonal difficulties (Vanheule et al., 2007), diminished self-efficacy (Calia et al., 2015; Faramarzi & Khafri, 2017; Lumley et al., 2002), lower self-esteem (Yelsma, 1995), and reduced social support (Di Tella et al., 2018; Karukivi et al., 2010). Particularly noteworthy is its association with pain catastrophizing (Lumley et al., 2002; Makino et al., 2013), which has been conceptualized as, among other things, a maladaptive means of communicating distress and eliciting interpersonal support (Mikulincer, 1998; Sullivan et al., 2001). Consequently, alexithymia may help account for several seemingly distinct cognitive, emotional, and interpersonal processes identified by the ADMCP, providing a potentially more parsimonious pathway through which attachment anxiety contributes to perceived pain burden.

## 2. Materials and Methods

### 2.1. Subjects

Participants were 119 undergraduate students (*M*_age_ = 19.46; 52.1% female; see Table 1 for further demographics) who were recruited through posters at the University of North Carolina Wilmington (UNCW), as well as the university’s psychology department participant pool. The sample was representative of typical undergraduates at UNCW, and participants were not specifically screened for pain disorders or chronic pain experience.

Tables provided by Fritz and MacKinnon (2007) suggested that 75 participants would be sufficient to detect a moderate-sized indirect effect with 0.8 power and a 0.05 alpha level. Effect size expectations were based upon the moderate-to-large associations demonstrated among our variables of interest earlier (Celikel & Saatcioglu, 2006; Martínez et al., 2015; P. J. Meredith et al., 2005). Although our main models of interest were simple mediation models, more complex models were also planned (e.g., parallel mediation, inclusion of covariates), thus we planned to recruit more than 75 participants. Our decision rule was to stop data collection after two academic semesters. This study was not preregistered (which can increase the possibility of analytic flexibility); however, all data was collected prior to analysis, is publicly available (see Data Availability Statement below), and confirmatory and exploratory hypotheses are made explicit (see Analysis Plan below). Participants received partial research credit for their Introduction to Psychology course. Written consent in accordance with the university’s Institutional Review Board was obtained for all participants.

### 2.2. Measures

*Perceptions of pain burden.* The West Haven-Yale Multidimensional Pain Inventory (WHYMPI; Kerns et al., 1985) is a well-validated, multidimensional measure of pain that is divided into three parts (with a total of 52 items and 12 subscales). Part 1 comprises the following five subscales: (1) perceived pain interference in daily functioning; (2) social support from significant others; (3) pain severity; (4) perceived life control; and (5) affective distress. Part 2 measures the perceived extent that respondents’ significant others display Solicitous, Distracting or Negative responses to their pain behaviors and complaints. Finally, Part 3 measures how frequently respondents engage in the following daily activities: Household Chores, Outdoor Work, Activities Away from Home, and Social Activities. Items are rated on a 7-point scale from 0 to 6, with anchors that differ according to the context of the item. For example, item 1 asks “Rate the level of your pain at the present moment,” with scale anchors ranging from 0 (no pain) to 6 (very intense pain), whereas item 2 asks “In general, how much does your pain problem interfere with your day-to-day activities?”, with scale anchors ranging from 0 (no interference) to 6 (extreme interference). For the purposes of the current study, a composite score was calculated by adding the scores from the pain interference, pain severity, and affective distress subscales, such that higher values indicated higher levels of perceived pain burden. These subscales were chosen due to their relationship with the subjective experience of pain, and past research supports similar use of these subscales (Harlacher et al., 2011; McKillop & Nielson, 2011; Stroud et al., 2000). Internal consistency for perceived pain burden in the current study was good (α = 0.81).

*Trait attachment.* The Experiences in Close Relationships Scale-Short Form (ECR-Short Form; Wei et al., 2007) is a self-report scale that assesses individual differences in anxious (6 items; e.g., “I need a lot of reassurance that I am loved by my partner”) and avoidant (6 items; e.g., “I try to avoid getting too close to my partner”) attachment across 12 items. Each item is rated on a 7-point scale (from 1 = “definitely not like me” to 7 = “definitely like me”). Responses were summed across items to form composites of attachment anxiety and avoidance, such that higher scores indicated higher levels of those traits. Internal consistency for attachment anxiety (α = 0.71) and avoidance (α = 0.76) were acceptable in the current study.

*Threat appraisal.* The Pain Appraisal Inventory (PAI; Unruh & Ritchie, 1998) is a self-report measure of cognitive pain appraisals and includes a total of 16 items and two subscales (threat and challenge scales; 8 items each). The current study focused on threat scores (e.g., “I feel controlled by the pain”). Items are rated on a 6-point scale from 1 (strongly disagree) to 6 (strongly agree) and were summed to form a composite score, such that higher values meant higher levels of threat appraisal. Internal consistency for the threat appraisal composite in the current study was excellent (α = 0.92).

*Alexithymia.* The Twenty-Item Toronto Alexithymia Scale (TAS-20; Bagby et al., 1994) is 20-item self-report measure that is used extensively in alexithymia research. It is composed of three subscales- the Difficulty Describing Feelings subscale (5-items; e.g., “It is difficult for me to find the right words for my feelings”), the Difficulty Identifying Feelings subscale (7-items; e.g., “I am often confused about what emotion I am feeling”), and the Externally-Oriented Thinking subscale (8-items; e.g., “I prefer to analyze problems rather than just describe them”). Items are rated on a 5-point scale from 1 (strongly disagree) to 5 (strongly agree), and the sum of responses to all 20 items represents the total alexithymia score. Internal consistency for the TAS in the current study was good (α = 0.81).

*Self-esteem.* The Rosenberg Self-Esteem Scale (RSES; Rosenberg, 1965) is a widely used 10-item instrument for measuring global self-esteem. Items (e.g., “I certainly feel useless at times”—reversed-scored) were scored on a 4-point response scale, ranging from 1 (strongly disagree) to 4 (strongly agree). Item responses were summed to form a composite score, such that higher values meant higher levels of self-esteem. Internal consistency for the RSES in the current study was good (α = 0.88).

*Rejection sensitivity.* The Rejection Sensitivity Questionnaire (RSQ; Downey & Feldman, 1996) is a self-report measure of individual differences in one’s anxious expectations for rejection by significant others. The RSQ includes 18 hypothetical social interactions involving potential rejection by a significant other (e.g., “You ask your partner to move in with you”). Respondents rate their anxiety regarding the outcome of each interaction, as well as the perceived likelihood that their significant other will respond with rejection. Anxiety scores are weighted (multiplied) by the expected likelihood of rejection and then averaged across the 18 situations. Internal consistency for the RSQ in the current study was acceptable (α = 0.79).

*Pain catastrophizing.* The Pain Catastrophizing Scale (PCS; Sullivan et al., 1995) is a 13-item self-report measure developed to assess pain-related catastrophic thinking among adults both with or without chronic pain. Items (e.g., “I become afraid that the pain will get worse”) are rated on a 5-point scale ranging from 0 (not at all) to 4 (all the time). Item responses were summed to form a composite score, such that higher values meant higher levels of pain catastrophizing. Internal consistency for the PCS in the current study was excellent (α = 0.94).

*Perceived social support*. The Multidimensional Scale of Perceived Social Support (MSPSS; Zimet et al., 1988) is a 12-item questionnaire (4 items per subscale) designed to measure perceptions of support from three sources: friends, family, and a significant other. Items (e.g., “My friends really try to help me”, “I can talk about problems with my family”, and “There is a special person who is around when I am in need”) are rated on a 7-point scale from 1 (very strongly disagree) to 7 (very strongly agree). Item responses were summed to form a composite score, such that higher values meant higher levels of perceived social support. Internal consistency for the MSPSS in the current study was excellent (α = 0.92).

*Problematic substance use.* The Rutgers Alcohol Problem Index (RAPI; White & Labouvie, 1989) is an 18-item self-administered screening tool for assessing problem drinking in adolescents and young adults. The measure’s instructions ask, “How many times did the following things happen to you while you were drinking alcohol or because of your alcohol use during the past six months?” Participants rated items (e.g., “Got into fights, acted bad, or did mean things”) by frequency of occurrence on a five-point scale ranging from 1 (never) to 5 (more than ten times). Item responses were summed to form a composite score, such that higher values meant higher levels of problematic substance use. Internal consistency for the RAPI in the current study was excellent (α = 0.91).

### 2.3. Procedure

In a single laboratory session, participants were directed to individual cubicles and were informed that the study was ostensibly investigating the relationship between personality and “physical sensations.” After providing consent, participants were asked to complete each of the measures previously described (please see Table 2 for descriptive statistics for each measure). Following completion of these measures, participants were debriefed and awarded course credit for participation.

### 2.4. Analysis Plan

Prior to formal analysis, the distributional properties of all continuous variables were examined empirically with descriptive statistics and visually inspected using histograms and box-plots. Model assumptions (e.g., linearity, normality, homogeneity of error variance) were examined with the use of q-q plots and residual plots. We also examined multicollinearity diagnostics (e.g., zero-order correlations, variance inflation factors). Violations were not indicated; furthermore, the influence of disproportionate outliers was not present. Thus, no remedial actions were required prior to proceeding with formal analysis. Negligible missing data were identified across items of primary interest (ECR-S, TAS-20, RAPI, PAI; <1% of data), which was addressed by substituting participants’ mean scores on each of the measures for the very few missing cases.

Several mediation models were tested, following the recommendations by Baron and Kenny (1986) and Preacher and Hayes (2008) and Hayes (2022). Mediation models were tested using ordinary least-squares multiple regression and Model 4 of Hayes (2022) Process Macro in IBM SPSS Statistics (Version 29; IBM Corp, Armonk, NY, USA). 95% bias-corrected and accelerated bootstrapped confidence intervals (Preacher & Hayes, 2008) were computed from 5000 bootstrapped resamples and used to test the significance of all indirect effects. Attachment anxiety was entered as the focal predictor variable of interest and perceived pain burden was entered as the focal outcome across all models. Simple mediation models were first tested to examine the indirect influence of threat appraisal and alexithymia, separately, on the association between attachment anxiety and pain burden. The predicted mediators were then allowed to compete simultaneously in a parallel mediation model. In a final, exploratory analysis, all measured variables relevant to the ADMCP were entered simultaneously in a post hoc parallel mediation model, in order to examine the independent contribution of each variable to the association between anxious attachment and pain burden. Unstandardized regression coefficients are reported for all model paths.

Finally, attachment anxiety and avoidance are each theoretically distinct, but often correlated, dimensions of attachment insecurity (Brennan et al., 1998; Fraley et al., 2000). Our hypotheses concerned the unique influence of attachment anxiety specifically, and not attachment insecurity more generally. Thus, despite weak associations with attachment anxiety (*r* = 0.04, *p* = 0.67) and pain burden (*r* = 0.19, *p* = 0.04), we included attachment avoidance as a covariate across all models. This is a common approach in the literature on adult attachment security (e.g., Fraley et al., 2013; Nielsen et al., 2017).

## 3. Results

In the first set of simple mediation models, analyses indicated a significant relationship between attachment anxiety and perceptions of pain burden (Path C: *B* = 0.071, *t* = 2.22, *p* = 0.028), as well as between attachment anxiety and our proposed mediator variables (Path A-threat: *B* = 0.261, *t* = 2.495, *p* = 0.014; Path A-alexithymia: *B* = 0.771, *t* = 4.212, *p* < 0.001) and our mediator variables and perceptions of pain burden (Path B-threat: *B* = 0.168, *t* = 7.089, *p* < 0.001; Path B-alexithymia: *B* = 0.052, *t* = 3.364, *p* = 0.001). Furthermore, bootstrapped confidence intervals (Preacher & Hayes, 2008) revealed significant indirect effects (*ab*) in both models (threat: *ab* = 0.044, 95% BCA CI: [0.0102, 0.0802]; alexithymia: *ab* = 0.040, 95% BCA CI: [0.0149, 0.0729]). Further still, neither model produced significant direct effects of attachment anxiety in the presence of the mediators (threat: *B* = 0.027; 95% BCA CI: [−0.0273, 0.0809]; alexithymia: *B* = 0.031; 95% BCA CI: [−0.0342, 0.0952]). Thus, each mediator fully accounted for the association between attachment anxiety and perceived pain burden in their respective model.

After providing evidence of threat appraisal’s and alexithymia’s respective mediational influence underlying the association between attachment anxiety and perceived pain burden, we conducted a parallel mediation analysis in which all four of these variables were included in the same model to determine the degree to which threat appraisal influences the attachment anxiety-pain burden relationship while holding alexithymia constant, and vice versa. As shown in Table 3, only threat appraisal predicted perceived pain burden. Threat appraisal demonstrated a significant indirect effect on the attachment anxiety-pain burden relationship (*ab* = 0.041, 95% BCA CI: [0.0089, 0.0779]). The indirect effect for perceived pain burden via alexithymia was not significant (*ab* = 0.014, 95% BCA CI: [−0.0095, 0.0437]). However, a contrast comparing the two indirect effects did not indicate a significant difference between the two potential mediators (*B* = −0.024, 95% BCA CI: [−0.0769, 0.0199]). Thus, although threat appraisal maintained a significant indirect effect with the inclusion of alexithymia and the indirect effect of alexithymia dropped from significance, the two indirect effects were not statistically different from each other. Last, the direct effect of attachment anxiety on perceived pain burden was not significant (*B* = 0.016, 95% BCA CI: [−0.0409, 0.0724]) when accounting for the mediators.

### Exploratory Mediation Analysis

We conducted a final mediation analysis to evaluate other mediator variables proposed by the ADMCP (P. Meredith et al., 2008) alongside threat appraisal and alexithymia. Based on the relative importance attributed by the ADMCP, we chose to examine pain catastrophizing, self-esteem, rejection sensitivity, perceived social support, and problematic substance use as additional potential mediators. Parallel mediation analyses revealed that both self-esteem (*ab* = 0.022, 95% BCA CI: [0.0006, 0.0477]) and threat appraisal (*ab* = 0.031, 95% BCA CI: [0.0062, 0.0650]) had significant indirect effects in this model (see Table 4). Lastly, the direct effect of attachment anxiety on perceived pain burden was not significant (*B* = −0.0164, 95% BCA CI: [−0.0735, 0.0407]).

## 4. Discussion

Our primary aim was to examine the relationship between attachment anxiety and perceived pain burden among healthy young adults using the ADMCP (P. Meredith et al., 2008) as the foundation for our predictions. The ADMCP is a complex model that has stimulated different streams of research (Andrews et al., 2014; Forsythe et al., 2012; Laird et al., 2015; Tremblay & Sullivan, 2010); however, this research has demonstrated that numerous variables mediate the relationship between attachment anxiety and pain, without providing greater insight into which of these variables may have the most explanatory power. We predicted that the association between attachment anxiety and perceived pain burden would best be explained by threat appraisal and alexithymia–two constructs closely related to anxious attachment style (Bartholomew et al., 1997; Berenbaum & James, 1994; Montebarocci et al., 2004; Porter et al., 2007).

Our findings provided partial support for the proposed mediation model. When examined independently, both threat appraisal and alexithymia significantly mediated the association between attachment anxiety and perceived pain burden, consistent with previous work implicating these processes in attachment-related pain experiences (P. J. Meredith et al., 2005; Peñacoba et al., 2018; Stamp et al., 2024; Zdankiewicz-Ścigała & Ścigała, 2018). A different pattern emerged, however, when their unique contributions were evaluated simultaneously. In the parallel mediation model, the indirect effect through threat appraisal remained statistically significant, whereas the indirect effect through alexithymia did not. This pattern suggests that some of the explanatory information provided by alexithymia, when considered independently, may overlap with that captured by threat appraisal. Nevertheless, the indirect effects themselves did not differ significantly from one another. Thus, the present findings do not establish threat appraisal as a stronger mediator; instead, they suggest that the unique contribution of threat appraisal was sufficient to remain detectable after accounting for variance shared by the two proposed mediators.

This overlap may reflect a meaningful conceptual connection between threat appraisal and alexithymia that was not fully considered in formulating our original hypotheses. Indeed, the two constructs were moderately correlated in the present study (*r* = 0.49, *p* < 0.001). The broader literature provides a theoretical basis for this association. Difficulties identifying and interpreting emotional experiences may contribute to perceiving emotions as globally threatening or unmanageable (Gross, 2015; Preece et al., 2022; Sifneos, 1973; Swart et al., 2009), and alexithymia has even been conceptualized as a learned strategy for avoiding unwanted emotional experiences (Panayiotou et al., 2015). From this perspective, alexithymia and threat appraisal may share an underlying tendency to perceive internal experiences as exceeding one’s capacity to effectively understand or manage them. Whereas threat appraisal in the present study reflected the perception that one lacks the resources necessary to meet the demands posed by pain (Lazarus & Folkman, 1984; Putwain & Symes, 2014, 2016), alexithymia may similarly reflect difficulties navigating the demands posed by emotional experiences. Given that emotional distress can itself be experienced as painful (Sachs-Ericsson et al., 2017), this conceptual overlap may help explain why alexithymia did not account for a significant unique indirect effect once threat appraisal was included in the model.

To further explicate the relationship between attachment anxiety and perceived pain burden, we conducted an exploratory, parallel mediation analysis that evaluated several variables central to the ADMCP (P. Meredith et al., 2008) as potential mediators alongside threat appraisal and alexithymia. Findings from this analysis indicated that threat appraisal and self-esteem significantly mediated the relationship between attachment anxiety and perceived pain burden. These results continue to support threat appraisal’s role in underlying the relationship between attachment anxiety and pain, while also drawing attention to self-esteem. However, given the exploratory, post hoc nature of this analysis, these results should be interpreted with caution.

The central role of threat appraisal in our findings is consistent with the basic premises of attachment theory. Internal working models—expectations, emotions, and behavioral strategies that guide responses to salient experiences—are thought to develop partly through early experiences involving threats to security (Bowlby, 1982). For individuals high in attachment anxiety, inconsistent caregiver responsiveness may contribute to heightened vigilance for potential threats (Mikulincer & Shaver, 2007), including a greater tendency to detect threat in ambiguous situations (Ein-Dor et al., 2011). This heightened threat sensitivity may subsequently promote maladaptive responses to stressful experiences (Berry & Kingswell, 2012; Bowlby, 1982; Green & Douglas, 2018; Mikulincer & Florian, 2000). Pain may be particularly susceptible to this process. Because pain inherently signals potential threat (Eccleston & Crombez, 1999), the heightened threat sensitivity associated with attachment anxiety (Chester et al., 2012; Mikulincer & Florian, 2000; Shannon & Pond, 2022) may predispose individuals to appraise painful experiences as exceeding their capacity to cope. Such appraisals could provide an early psychological pathway through which attachment-related vulnerability contributes to maladaptive responses to pain and, potentially, its persistence over time (Stamp et al., 2024).

Our exploratory findings raise the possibility that self-esteem may represent one process through which these threat appraisals are translated into greater pain burden. We initially focused on alexithymia because difficulties identifying, interpreting, and communicating emotional and somatic experiences offered a parsimonious explanation for the ineffective coping frequently associated with attachment anxiety. Moreover, alexithymia is related to several psychological processes identified within the ADMCP, including lower self-esteem (Yelsma, 1995). The present findings, however, suggest that self-esteem itself may deserve greater attention as a potential mechanism. Low self-esteem has been associated with several forms of maladaptive coping implicated in the ADMCP (Dorard et al., 2013; Ikiz & Cakar, 2010; Lee et al., 2014), and theoretical accounts have proposed that repeated perceptions of stressors as threatening or unmanageable may undermine individuals’ evaluations of their own competence and worth (Bednar & Peterson, 1995; Dysvik et al., 2005). Viewed from this perspective, our post hoc findings suggest a plausible model in which attachment anxiety is associated with heightened perceptions of pain-related threat and diminished self-esteem, which are subsequently associated with greater perceived pain burden.

One reason self-esteem may be particularly relevant to pain adjustment is its potential influence on adaptive problem-solving and coping. Low self-esteem has been associated with poorer social problem-solving—the purposeful, self-directed process through which individuals attempt to navigate everyday social challenges (Chang & D’Zurilla, 1996; D’Zurilla & Goldfried, 1971; Hamarta, 2009; Koruklu, 2015). Effective problem-solving, in contrast, is conceptually inconsistent with the passive and maladaptive coping responses emphasized by the ADMCP and has been associated with reduced pain (Suso-Ribera et al., 2016). Self-esteem may, therefore, reflect a relatively broad psychological resource that influences whether individuals perceive themselves as capable of responding effectively to pain and other stressors. This interpretation also suggests a potential refinement of our original model: rather than alexithymia alone accounting for deficient coping among individuals high in attachment anxiety, threat appraisal may undermine broader evaluations of personal coping capacity, including self-esteem, which may influence subsequent pain-related responses. Because this pathway emerged from post hoc analyses, however, it should be regarded as hypothesis-generating and tested directly in future research.

### 4.1. Limitations and Future Directions

Our study had a number of limitations to consider. First, our study’s sample was nonclinical and composed mostly of white undergraduate students, of whom more than half were female, who were relatively healthy. This impacts the generalizability of our results. We know that patterns in the psychological literature can vary substantially across populations (Henrich et al., 2010). Although our results were in line with our predictions and expectations based upon the ADMCP, they should still be interpreted with caution. Future research should aim to replicate these results across a variety of different sample populations.

Second, our findings are limited by the cross-sectional, correlational, and self-report nature of the research design. Our findings may not reveal directional or causal relationships. It is unclear whether attachment anxiety induces heightened levels of threat appraisal and alexithymia and reduces self-esteem, which then induces pain-related symptoms. The directional nature of the variables may be in an alternative order, which cross-sectional, correlational designs do not preclude. For instance, perhaps pain-related experiences activate attachment processes? Furthermore, an unmeasured third variable (e.g., depression, anxiety, pain self-efficacy, negative affect) may fully account for the patterns of covariation observed among our variables of interest. For instance, it could be the case that attachment-related distress induces negative affect, which then mutually influences both pain-related symptoms and appraisal. Our present study cannot account for these alternative, competing models. In addition, common method variance could be a concern, as every construct was measured through self-report, and, thus, shared response tendencies could potentially account for the pattern of relationships among the variables of interest in our models (Podsakoff et al., 2003). Future work should employ longitudinal and experimental designs using a combination of behavioral and self-report measures to further examine the directional and causal relationships among these variables.

Third, although we were adequately powered for our simple mediation models, it is possible that we were underpowered for the parallel mediation models. Thus, the lack of a unique, significant indirect effect for alexithymia (while entered simultaneously with threat appraisal) should be interpreted with caution. Furthermore, the larger parallel mediation model with multiple ADMCP variables included as potential mediators should also be interpreted with caution, particularly given its post hoc nature. The lack of a significant indirect effect in this context does not necessarily mean that those variables would not exhibit an indirect effect in a similar model for a future study.

Last, our measures focused on variables central to the ADMCP. However, there would be value in including different mood measures in the future. Research suggests that low self-esteem predicts both depression and anxiety (Sowislo & Orth, 2013), and that the latter variables predict pain-related symptoms (Tegethoff et al., 2015). Given these connections, as well as similar links between anxious attachment and depression/anxiety (Jinyao et al., 2012), the inclusion of mood measures would further clarify associations.

### 4.2. Implications

Despite this study’s limitations, its results contribute in a novel fashion to the literature by both simplifying and adding precision to extant explanations of the relationship between attachment anxiety and the pain experience. Though our primary mediation hypothesis was only partially confirmed, our findings provided valuable insight into the variables that account for the perceived pain burden among people with heightened attachment anxiety. The identification of threat appraisal, alexithymia, and self-esteem as potential mediators of this relationship is a critical step to future experimental work that may lead to the development of targeted interventions aimed at reducing pain among people with heightened attachment anxiety.

For example, given our findings concerning self-esteem, people with heightened attachment anxiety and a pain condition may possibly benefit from interventions designed to bolster their sense of self. The field of psychology counts numerous interventions designed to aid individuals with low self-esteem, including cognitive-behavioral interventions, reminiscence-based interventions (focused on helping individuals recover positive autobiographic memories and then reflect on their content), support groups, and art therapy (Niveau et al., 2021). Work within the self-esteem literature suggests that self-esteem may have an anxiety-buffering function (Greenberg et al., 1992). Although much more experimental work is needed, it is not entirely unreasonable to believe that targeting anxiously attached individuals’ self-esteem could help reduce their pain experience by also reducing anxiety and minimizing the degree to which they appraise stimuli as threatening.

## 5. Conclusions

Given the physical, psychological, and economic burdens associated with the experience of pain (Gaskin & Richard, 2012; Institute of Medicine, 2011), continued efforts need to be made to identify ways of reducing pain symptomatology among its sufferers. Research suggests that attachment anxiety figures prominently in the experience of pain, and we thus endeavored to better understand the factors that may predispose people to pain as a function of attachment anxiety. Though the current study has helped shed further light on critical aspects of the association between attachment anxiety and pain burden, we encourage future research that attempts to replicate and expand upon our work.

## Figures and Tables

**Table 1:** Demographic Characteristics of Sample.

Characteristic	Category	Frequency	Percent
Gender	Male	53	44.5
	Female	62	52.1
	Non-Binary	3	2.5
	Self-Described	1	0.8
Ethnicity	Caucasian	88	73.9
	African American	7	5.9
	Latino/Hispanic	11	9.2
	Asian	2	1.7
	Two or More	8	6.7
	Other/Unknown	3	2.5
Education	High School	114	95.8
	Bachelor’s	4	3.4
	Trade School	1	0.8
Employment	Employed F/T	6	5
	Employed P/T	56	47.1
	Seeking Opportunities	54	45.4
	Retired	1	0.8
	Prefer Not to Say	2	1.7
Marital	Single (Never Married)	108	90.8
	Married	3	2.5
	Domestic Partnership	7	5.9
	Divorced	1	0.8

*Note. N* = 119.

**Table 2:** Sample Descriptives of Study Variables.

Variable	*N*	*M*	*SD*	α
COMP PB	119	4.42	2.66	0.81
ANX-ATT	119	22.76	7.48	0.71
AVO-ATT	119	17.42	7.38	0.76
TAS-20	119	61.51	16.65	0.81
RAPI	119	8.66	10.36	0.91
THREAT	119	19.53	8.78	0.92
PCS	119	13.82	11.18	0.94
RSES	119	30.72	5.03	0.88
RSQ	119	8.10	3.73	0.79
MSPSS	119	66.69	13.36	0.92

*Note.* COMP PB = Composite Pain Burden; ANX-ATT = Anxious attachment; AVO-ATT = Avoidant-Attachment; TAS-20 = Alexithymia; RAPI = Problematic Substance Use; THREAT = Threat Appraisal; PCS = Pain Catastrophizing; RSES = Self-Esteem; RSQ = Rejection Sensitivity; MSPSS = Perceived Social Support; *M* = Mean; *SD* = Standard Deviation; α = Cronbach’s alpha.

**Table 3:** Summary of Parallel Mediation Analysis.

Total Effect (Anx → PB)	Direct Effect (Anx → PB)	Relationship	Indirect Effect	95% BCA CI
0.071 (0.028)	0.016 (0.583)	Anx → Threat → PB	0.040	[0.0089, 0.0779]
		Anx → Alex → PB	0.014	[−0.0095, 0.0437]

*Note. N* = 119; Parenthetical Values = *p* value; Anx = Attachment Anxiety; PB = Pain Burden; THREAT = Threat Appraisal; Alex = Alexithymia; BCA CI = Bias-corrected and accelerated confidence intervals.

**Table 4:** Summary of Final Parallel Mediation Analysis.

Total Effect (Anx → PB)	Direct Effect (Anx → PB)	Relationship	Indirect Effect	95% BCA CI
0.071 (0.028)	−0.016 (0.571)	Anx → Threat → PB	0.031	[0.0062, 0.0650]
		Anx → Alex → PB	−0.002	[−0.0084, 0.0470]
		Anx → RAPI → PB	0.005	[−0.0089, 0.0193]
		Anx → RSES → PB	0.022	[0.0006, 0.0477]
		Anx → PCS → PB	0.017	[−0.0004, 0.0415]
		Anx → MSPSS → PB	−0.001	[−0.0095, 0.0064]
		Anx → RSQ → PB	0.014	[−0.0112, 0.0454]

*Note. N* = 119; Parenthetical Values = *p* value; Anx = Attachment Anxiety; PB = Pain Burden; THREAT = Threat Appraisal; Alex = Alexithymia; RAPI = Problematic Substance Use; PCS = Pain Catastrophizing; RSES = Self-Esteem; RSQ = Rejection Sensitivity; MSPSS = Perceived Social Support; BCA CI = Bias-corrected and accelerated confidence intervals.

## Data Availability

All data for the manuscript can be found on the Open Science Framework (at: https://osf.io/uzwvd/?view_only=b6cfb16b927f40f0bcd730d22e2d36ad (accessed on 12 June 2024)).
